# Early Real-World Clinical Outcomes and an Exploratory Cost-Impact Projection of Paclitaxel Drug-Coated Balloon Dilatation for Recurrent Male Urethral Stricture Disease: A Single-Centre Retrospective Study

**DOI:** 10.7759/cureus.115157

**Published:** 2026-08-25

**Authors:** Sami Atiq, Ahmed Anber, Praveen Subbiah, Wasim Mahmalji

**Affiliations:** 1 Urology, Hereford County Hospital, Wye Valley NHS Trust, Hereford, GBR; 2 Urology, Barking Havering and Redbridge NHS Trust, London, GBR; 3 Urology, Cambridge University Hospitals NHS Trust, Cambridge, GBR; 4 Urology, Wye Valley NHS Trust, Hereford, GBR

**Keywords:** cost-impact projection, drug-coated balloon, endoscopic treatment, paclitaxel, real-world outcomes, urethral stricture, uroflowmetry

## Abstract

Objective

This study aimed to evaluate changes in the International Prostate Symptom Score (IPSS) as the primary functional outcome following Optilume drug-coated balloon dilatation for recurrent male urethral stricture disease. Secondary outcomes included maximum urinary flow rate, post-void residual (PVR) volume, patient satisfaction, adverse events, and repeat intervention. An exploratory manufacturer-supplied cost-impact projection was also described separately.

Materials and methods

This retrospective single-centre study included 10 men treated during the early implementation of the Optilume drug-coated balloon (Urotronic, Inc., Plymouth, MN). Routinely recorded IPSS, maximum urinary flow rate (Qmax), PVR, satisfaction, complications, and repeat intervention data were reviewed. An exploratory cost-impact projection was generated using a manufacturer-supplied model and interpreted alongside economic guidance from the National Institute for Health and Care Excellence (NICE). This illustrative single-year projection was not a formal budget-impact analysis and was not based on audited, patient-level local cost data.

Results

The mean age of the cohort was 62.5 ± 6.5 years. All 10 patients (100%) had bulbar urethral strictures. Median IPSS decreased from 15.0 (interquartile range (IQR): 13.3-16.0) before treatment to 2.0 (IQR: 2.0-3.5) at follow-up (Wilcoxon signed-rank test, W = 1, p = 0.0039). Median Qmax increased from 7.5 (IQR: 5.3-9.0) mL/s to 18.8 (IQR: 15.4-19.8) mL/s (W = 0, p = 0.0020), and median PVR decreased from 211.5 (IQR: 150.0-239.0) mL to 20.0 (IQR: 11.3-26.8) mL (W = 0, p = 0.0020). Nine of 10 patients reported satisfaction with treatment. No urinary tract infection, urinary retention, haematuria requiring intervention, unplanned admission, or repeat stricture procedure was recorded at the latest follow-up. In the manufacturer-supplied model for 30 annual procedures, projected initial-procedure expenditure was £11,430 lower with Optilume after applying an assumed £1,500 reimbursement. Projected retreatment expenditure was approximately £17,252 lower. These figures were model-derived outputs and did not represent observed or audited local savings.

Conclusions

Drug-coated balloon dilatation was associated with early symptomatic and objective improvement in this selected cohort, although the uncontrolled study design does not allow conclusions regarding causation, comparative effectiveness, or durability. No major adverse events were documented, but the cohort was insufficient to assess uncommon complications. The exploratory cost-impact projection illustrates possible expenditure differences under the stated assumptions but does not demonstrate actual or realised savings.

## Introduction

Recurrent male urethral stricture disease remains difficult to manage because an initially successful endoscopic opening may be followed by recurrent fibrosis and restenosis. Dilatation and direct visual internal urethrotomy are accessible and minimally invasive, but repeated procedures have limited durability, whereas urethroplasty offers a more durable repair at the cost of greater invasiveness and the need for a reconstructive pathway. The OPEN randomised trial confirmed that both urethrotomy and urethroplasty improve symptoms in recurrent bulbar disease, while urethroplasty is associated with fewer subsequent reinterventions [1].

The Optilume drug-coated balloon (Urotronic, Inc., Plymouth, MN) combines mechanical dilatation with local delivery of paclitaxel, an antiproliferative agent intended to reduce fibroblast-mediated scar recurrence. The National Institute for Health and Care Excellence (NICE) recommends Optilume as an option for recurrent bulbar urethral strictures provided that comparative patient-reported outcome and reintervention data are collected; the technology is intended for strictures 3 cm or less in length after at least one failed endoscopic procedure [2]. Contemporary American Urological Association and European Association of Urology guidance similarly position drug-coated balloon treatment as a selected option for short, recurrent bulbar strictures in men who wish to avoid, or are not suitable for, urethroplasty [3,4].

Randomised and prospective evidence from the ROBUST programme has shown improved anatomical patency and reduced repeat intervention compared with standard endoscopic management, with follow-up extending to three to five years [5-8]. However, UK real-world data from district general hospital practice remain limited, and local financial consequences depend on procedural costs, reimbursement, and retreatment rates. This study evaluated early functional outcomes, patient satisfaction, safety, and retreatment after the introduction of the technology at a single UK centre and included a separate exploratory cost-impact projection.

## Materials and methods

Study design and setting

We performed a retrospective, single-centre observational study of men who underwent paclitaxel drug-coated balloon dilatation for recurrent urethral stricture disease at Hereford County Hospital, Wye Valley NHS Trust, between June 1, 2024, and June 30, 2026. The study used routinely collected clinical data and was reported in accordance with the Strengthening the Reporting of Observational Studies in Epidemiology (STROBE) statement [9].

Participants

Men were eligible if they underwent drug-coated balloon dilatation for symptomatic recurrent anterior urethral stricture disease measuring no more than 3 cm after at least one previous endoscopic procedure and had paired pre-treatment and follow-up functional outcomes available. Patients with active urinary infection, long or obliterative disease, dense lichen sclerosus-related disease, complex previous reconstruction, or another contraindication to the device were not considered suitable candidates. Ten patients underwent treatment during the study period; all 10 had complete paired functional outcomes and were included in the analysis. No treated patients were excluded from the analysis.

Preoperative assessment and procedure

**Figure 1:**
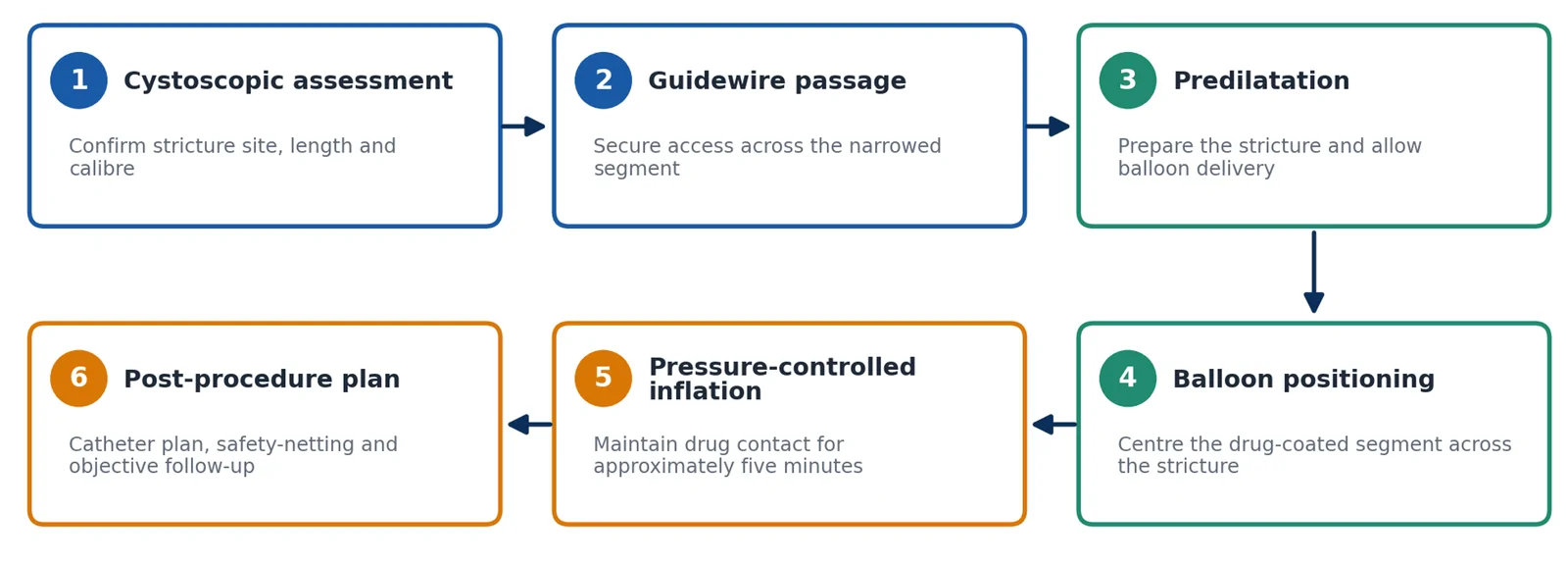
Procedural workflow for Optilume drug-coated balloon dilatation The procedural workflow comprised cystoscopic assessment of the stricture, passage of a guidewire, predilatation, centring of the drug-coated balloon across the diseased segment, pressure-controlled inflation for approximately five minutes, and postoperative catheter and follow-up planning Original figure created by the authors

Baseline evaluation comprised clinical history, previous stricture treatment, symptom assessment, uroflowmetry, and bladder scanning where available, together with cystoscopic assessment and/or urethral imaging according to clinical need. Urine infection was treated before instrumentation. The stricture was assessed cystoscopically, a 0.035-inch guidewire was passed across the narrowed segment, and predilatation was undertaken. The 5 cm drug-coated balloon was centred across the stricture and inflated with a pressure-controlled device for approximately five minutes. Continuous irrigation was avoided during the drug-contact phase where possible. Procedures were performed under general anaesthesia, followed by a same-day trial without a catheter according to local practice. The procedural sequence is summarised in Figure 1.

Clinical outcomes

The primary functional outcome was change in International Prostate Symptom Score (IPSS) from baseline to the recorded postoperative assessment. Secondary outcomes were changes in Qmax and post-void residual (PVR), patient-reported satisfaction, treatment-related complications, unplanned healthcare use, and repeat intervention. Satisfaction was abstracted from routine postoperative clinical documentation as a binary yes/no outcome. The wording and timing of the assessment were not standardised, and no validated satisfaction instrument was used. The IPSS is a validated seven-item symptom scale scored from 0 to 35 [10]. Standard severity categories were used: mild: 0-7; moderate: 8-19; and severe: 20-35. Outcomes were obtained from the latest documented postoperative assessment available during the study period. Follow-up was not undertaken at a uniform prespecified time point and ranged from four to 12 months; therefore, the postoperative measurements represent variable-duration follow-up rather than outcomes assessed at a fixed time point.

Exploratory cost-impact projection

Because patient-level local costing and mature observed retreatment data were not available, neither a formal cost-effectiveness analysis nor a formal budget-impact analysis was undertaken. Instead, an illustrative single-year cost-impact projection was generated using a manufacturer-supplied Wye Valley model (Laborie Medical Technologies, 2026; unpublished). The model assumed 30 theatre-based procedures per year, a theatre cost of £600 for 30 minutes, Optilume device and consumable costs of £1,550, standard endoscopic consumable costs of £431, and an assumed reimbursement of £1,500 for each Optilume procedure. The £1,500 reimbursement was a model input rather than a universally applicable tariff, and reimbursement and coverage arrangements may differ substantially between healthcare systems and countries.

The resulting gross Optilume procedural cost was £2,150 per case, with a modelled net cost of £650 after reimbursement, compared with £1,031 for standard endoscopic care. The manufacturer-supplied model projected 18.7 retreatments following standard endoscopic care and 4.1 following Optilume within 12 months. The underlying retreatment denominator and calculations were not independently validated against observed local retreatment data. The model was unpublished and proprietary. Although the available inputs and outputs are reported, the complete internal formulas, population assumptions, and derivation of the projected retreatment values were not independently available for verification or reproduction. The projection was not validated against Wye Valley NHS Trust finance data, procurement records, or observed local retreatment rates. It used an assumed annual volume of 30 procedures and did not incorporate local epidemiological population sizing, an uptake trajectory, discounting, or sensitivity analysis.

NICE economic guidance was used to provide external context. NICE lists the single-use Optilume device at £1,350 excluding value-added tax and concluded that the technology was likely to be cost-saving over five years compared with standard care, while highlighting uncertainty regarding long-term recurrence [2]. A peer-reviewed NHS economic evaluation reported estimated total five-year costs of £9,122 for standard endoscopic management and £6,620 for Optilume [11].

Statistical analysis

Statistical analysis was performed using Python version 3.13.5 and SciPy version 1.17.0. Age is presented as mean ± standard deviation (SD), functional outcomes are presented as medians with interquartile range (IQR), and categorical variables as counts and percentages. Because IPSS and PVR are bounded at zero and their follow-up distributions demonstrated floor effects and right-skewness, paired pre-treatment and follow-up functional measurements were compared using two-sided Wilcoxon signed-rank tests. Exact two-sided p-values are reported. Paired-samples t-tests were retained as sensitivity analyses and produced concordant conclusions. A p-value < 0.05 was considered statistically significant. Given the small exploratory cohort, no multivariable analyses were performed. The cost projection was analysed descriptively because it was generated from an assumption-based, manufacturer-supplied cost-impact model.

## Results

Cohort characteristics

Ten patients with paired functional outcomes were included. The mean age was 62.5 ± 6.5 years. Patients underwent treatment between June 1, 2024, and June 30, 2026, with postoperative follow-up ranging from four to 12 months. All 10 patients (100%) had bulbar urethral strictures. Stricture length ranged from 2 to 3 cm, and none of the patients had previously undergone urethroplasty (Table 1).

**Table 1:** Cohort and procedural characteristics SD: standard deviation

Characteristic	Finding
Patients analysed, n	10
Age, years, mean ± SD	62.5 ± 6.5
Study period	June 1, 2024–June 30, 2026
Follow-up, months, range	4–12
Bulbar stricture, n (%)	10 (100%)
Stricture length, cm, range	2–3
Previous urethroplasty, n (%)	0 (0%)
Guidewire size	0.035 inch
Drug-coated balloon length, cm	5
Predilatation	Performed
Balloon inflation duration	Approximately 5 minutes
Anaesthesia	General anaesthesia
Postoperative catheter management	Same-day trial without catheter

Functional and patient-reported outcomes

**Figure 2:**
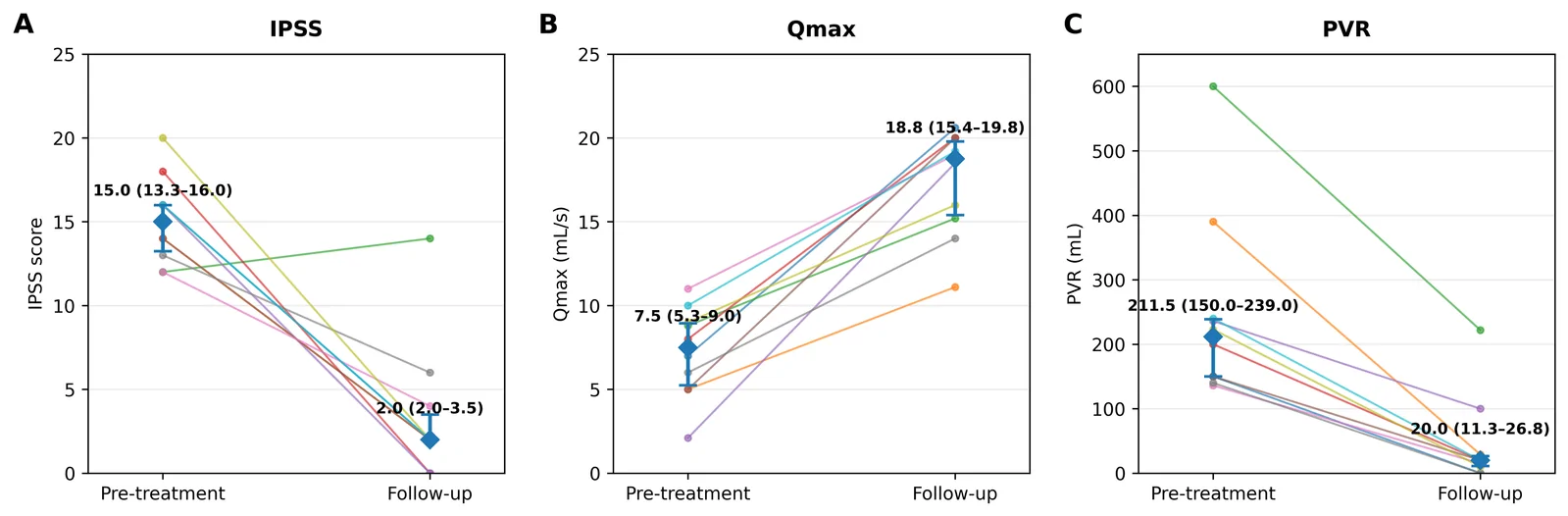
Pre-treatment and follow-up functional outcomes following Optilume drug-coated balloon dilatation Panel A shows the IPSS, which decreased from a median of 15.0 (IQR: 13.3–16.0) before treatment to 2.0 (IQR: 2.0–3.5) at follow-up; two-sided Wilcoxon signed-rank test, W = 1, p = 0.0039. Panel B shows maximum urinary flow rate (Qmax), which increased from 7.5 (IQR: 5.3–9.0) mL/s to 18.8 (IQR: 15.4–19.8) mL/s; two-sided Wilcoxon signed-rank test, W = 0, p = 0.0020. Panel C shows PVR, which decreased from 211.5 (IQR: 150.0–239.0) mL to 20.0 (IQR: 11.3–26.8) mL; two-sided Wilcoxon signed-rank test, W = 0, p = 0.0020. Thin lines connect individual paired observations. Central markers and whiskers represent cohort medians and IQRs IPSS: International Prostate Symptom Score; IQR: interquartile range; Qmax: maximum urinary flow rate; PVR: post-void residual volume Figure created by the authors from anonymised study data

All three functional outcomes improved significantly after treatment (Figures 2A-2C). Median IPSS decreased from 15.0 (IQR: 13.3-16.0) before treatment to 2.0 (IQR: 2.0-3.5) at follow-up (two-sided Wilcoxon signed-rank test, W = 1, p = 0.0039). Median Qmax increased from 7.5 (IQR: 5.3-9.0) mL/s to 18.8 (IQR: 15.4-19.8) mL/s (W = 0, p = 0.0020). Median PVR decreased from 211.5 (IQR: 150.0-239.0) mL to 20.0 (IQR: 11.3-26.8) mL (W = 0, p = 0.0020). Paired-samples t-test sensitivity analyses yielded concordant results: IPSS, t(9) = 6.09, p = 0.00018; Qmax, t(9) = 8.51, p = 0.000013; and PVR, t(9) = 6.81, p = 0.000078. Nine of 10 patients (90%) reported being satisfied with the treatment.

Safety and retreatment

No major treatment-related adverse events were documented during the available follow-up. Specifically, there were no documented urinary tract infections, episodes of urinary retention, haematuria requiring intervention, unplanned admissions, repeat endoscopic procedures, or urethroplasties. However, the absence of observed events in this cohort of 10 patients does not exclude uncommon complications; using the rule of three, the approximate upper 95% confidence bound for the true event rate is 30%.

Exploratory cost-impact projection

In the manufacturer-supplied cost-impact model, the gross Optilume procedural cost was £2,150 per case, comprising £600 for theatre time and £1,550 for the device and consumables. After applying the model’s assumed £1,500 reimbursement, the modelled net procedural cost was £650 per Optilume case. The modelled cost of standard endoscopic care was £1,031 per case, comprising £600 for theatre time and £431 for consumables. Under these assumptions, the modelled initial procedural cost was £381 lower per case with Optilume.

For an illustrative volume of 30 initial procedures per year, the model projected expenditure of £30,930 for standard endoscopic care and £19,500 for Optilume, representing a £11,430 lower projected expenditure with Optilume. Separately, the manufacturer-supplied cost-impact model projected 18.7 retreatments following standard endoscopic care and 4.1 following Optilume within 12 months, corresponding to 14.6 fewer repeat procedures and approximately 7.3 fewer theatre hours in the model. Projected retreatment expenditure was £20,073 following standard endoscopic care and £2,820 following Optilume, representing approximately £17,252 lower projected retreatment expenditure with Optilume. The initial-procedure and retreatment expenditure differences are reported separately and were not combined into a single annual saving. These figures represent illustrative outputs of the manufacturer-supplied cost-impact model rather than observed or independently audited local savings and therefore require independent validation (Table 2).

**Table 2:** Illustrative single-year cost-impact projection for 30 annual procedures

Model item	Standard endoscopic care	Optilume	Modelled difference
Gross procedural cost per case	£1,031	£2,150	Optilume, £1,119 higher before reimbursement
Assumed reimbursement per case	£0	£1,500	Manufacturer-model assumption
Net procedural cost per case	£1,031	£650	£381 lower with Optilume
Number of initial procedures per year	30	30	—
Net expenditure for 30 initial procedures	£30,930	£19,500	£11,430 lower with Optilume
Assumed retreatments within 12 months	18.7	4.1	14.6 fewer retreatments with Optilume
Approximate theatre time used for retreatment	9.35 hours	2.05 hours	Approximately 7.3 hours released
Projected retreatment expenditure	£20,073	£2,820	Approximately £17,252 lower with Optilume

## Discussion

This early single-centre study found substantial improvement in symptoms, urinary flow, and bladder emptying after drug-coated balloon dilatation in a selected cohort with recurrent urethral stricture disease. Median IPSS decreased from 15.0 to 2.0, median Qmax increased from 7.5 to 18.8 mL/s, and median PVR decreased from 211.5 to 20.0 mL. All three comparisons were statistically significant using two-sided Wilcoxon signed-rank tests, and paired-samples t-test sensitivity analyses yielded concordant results. The concordant improvement across a patient-reported measure and two objective functional measures supports a clinically encouraging early treatment signal. However, the small retrospective cohort and limited follow-up mean that the findings should be regarded as exploratory rather than confirmatory. Because this was an uncontrolled single-arm study, the observed changes cannot establish causation and may partly reflect regression to the mean or other unmeasured factors. The study also cannot determine comparative effectiveness against standard endoscopic treatment or urethroplasty, or establish long-term treatment durability.

The findings are directionally consistent with the ROBUST programme. ROBUST III showed higher six-month anatomical success and lower repeat intervention after drug-coated balloon treatment than after standard dilatation or urethrotomy in recurrent anterior strictures shorter than 3 cm [5]. Two-year and three-year follow-up demonstrated sustained symptom and retreatment outcomes in the intervention cohort [6,7]. ROBUST I reported an estimated 71.7% freedom from repeat intervention at five years in recurrent short bulbar strictures [8]. A 2026 multicentre real-world series of 319 patients reported one-year functional recurrence-free survival of 78.4% for anterior strictures, while showing poorer outcomes in some higher-risk subgroups [12]. Our series is too small, and follow-up is too early to estimate durability, but it provides a UK implementation signal based on routinely used functional measures.

Clinical positioning is important. Drug-coated balloon dilatation should not be presented as equivalent to urethroplasty. It is best considered an intermediate endoscopic option for carefully selected short recurrent bulbar strictures, particularly when a patient wishes to defer reconstruction or is not suitable for it. The 2026 European Association of Urology guideline recommends drug-coated balloon dilatation for short bulbar strictures recurring after at least two previous endoscopic treatments in patients who are unfit for, or unwilling to undergo, urethroplasty [4]. NICE recommends Optilume only when comparative patient-reported outcome and reintervention data are collected [2]. This makes systematic local follow-up part of safe implementation, rather than merely an optional audit exercise.

The exploratory cost-impact projection should be interpreted separately from the clinical outcomes. This was not a formal budget-impact analysis: it used a single-year snapshot and an assumed annual procedure volume and did not incorporate local population sizing, an uptake trajectory, discounting, or sensitivity analysis. The manufacturer-supplied model suggested a lower net initial procedural cost for Optilume only after applying the assumed £1,500 reimbursement. For 30 annual procedures, the projected initial-procedure expenditure difference was £11,430. Separately, projected retreatment expenditure was approximately £17,252 lower because the model projected 14.6 fewer repeat procedures. These figures were not observed local savings and were not combined into a single claimed annual benefit.

The projected expenditure differences were not derived from the observed clinical outcomes or patient-level financial data in this cohort and therefore cannot be interpreted as evidence of realised cost savings. NICE similarly concluded that Optilume was likely to be cost-saving over five years but highlighted uncertainty regarding long-term recurrence [2]. The peer-reviewed economic evaluation reported an estimated £2,502 lower cost per patient over five years compared with standard endoscopic management [11]. Because the local model was manufacturer-supplied, independent confirmation of the reimbursement pathway, procedural costs, and retreatment assumptions by local finance and procurement teams is required before conclusions regarding local cost impact can be made. The projected cost differences are specific to the modelled UK setting and should not be extrapolated to other healthcare systems, where device pricing, theatre costs, coverage, and reimbursement arrangements may differ substantially.

The current dataset also highlights practical governance priorities. Baseline stricture length, site, aetiology, and previous procedures should be recorded consistently. Postoperative IPSS, Qmax with voided volume, PVR, adverse events, and satisfaction should be collected at prespecified intervals, with repeat intervention treated as a key durability endpoint. A prospective registry at three, six, 12, and 24 months would allow survival analysis, clearer subgroup description, validation of costs, and benchmarking against trial and real-world series.

Limitations

This study has several limitations. It was a retrospective, single-centre study involving only 10 patients and did not include a concurrent comparator group, limiting statistical power, generalisability, and the ability to draw comparative conclusions. The uncontrolled design prevents attribution of the observed improvements solely to treatment, does not exclude regression to the mean, and does not permit conclusions regarding comparative effectiveness against standard endoscopic treatment or urethroplasty. Selection bias was possible because treatment was offered to patients considered suitable for drug-coated balloon dilatation.

Procedures were performed by more than one surgeon within routine clinical practice, and potential inter-operator variation in stricture assessment, predilatation, balloon positioning, and perioperative decision-making was not formally evaluated; this may have influenced the observed outcomes. Follow-up varied from four to 12 months and was insufficient to establish long-term durability or late retreatment rates. The follow-up IPSS and PVR distributions demonstrated floor effects and right-skewness; therefore, mean ± standard deviation alone did not adequately describe the distributions, and functional outcomes were presented using medians and interquartile ranges with Wilcoxon signed-rank testing. Patient satisfaction was recorded as a non-validated binary outcome. No major adverse events were observed, but the occurrence of zero events in a cohort of 10 patients does not exclude uncommon harms; using the rule of three, the approximate upper 95% confidence bound for the true event rate is 30%.

In addition, the cost component was an illustrative manufacturer-supplied cost-impact projection rather than a formal budget-impact analysis. It was not based on audited patient-level local cost data, did not include local population sizing, uptake over time, discounting, or sensitivity analysis, and could not be fully reproduced from the unpublished proprietary model. The projected expenditure differences should therefore not be interpreted as evidence of realised local savings.

## Conclusions

In this early single-centre experience, paclitaxel drug-coated balloon dilatation was associated with statistically significant improvements in IPSS, Qmax, and PVR in selected men with recurrent urethral stricture disease. However, the small, retrospective, single-arm, and uncontrolled design cannot establish causation, comparative effectiveness, or long-term durability. No major complications or repeat stricture procedures were documented during the available follow-up, but uncommon adverse events cannot be excluded. The exploratory manufacturer-supplied cost-impact projection illustrates possible expenditure differences under the stated reimbursement and retreatment assumptions but does not demonstrate observed or realised cost savings. Larger prospective comparative cohorts with standardised follow-up, complete retreatment reporting, and independently validated patient-level costing are required before firm conclusions can be drawn regarding effectiveness, safety, durability, or local cost impact.
